# Testing the role of the posterior cingulate cortex in processing salient stimuli in cannabis users: an rTMS study

**DOI:** 10.1111/ejn.14194

**Published:** 2018-10-27

**Authors:** Shikha Prashad, Elizabeth S. Dedrick, Wing Ting To, Sven Vanneste, Francesca M. Filbey

**Affiliations:** ^1^ Center for BrainHealth School of Behavioral and Brain Sciences University of Texas at Dallas 2200 West Mockingbird Lane Dallas TX 75235 USA

**Keywords:** EEG ERP, exteroceptive processes, neuromodulation, posterior cingulate cortex, precuneus

## Abstract

The posterior cingulate cortex (PCC) and precuneus are hubs in the default mode network and play a role in processing external salient stimuli. Accordingly, activation in these regions has been associated with response to salient stimuli using drug cue‐reactivity paradigms in substance using populations. These studies suggest that the PCC and precuneus may underlie deficits in processing salient stimuli that contribute toward the development of substance use disorders. The goal of this study was to directly test this hypothesis using repetitive transcranial magnetic stimulation (rTMS). Using a double‐blind, placebo‐controlled design, we used rTMS to target the PCC and precuneus with a double‐cone coil at 10 Hz (high frequency) and 1 Hz (low frequency) in 10 adult cannabis users and 10 age‐ and sex‐matched non‐using controls. Electroencephalography data were collected before and after rTMS during a modified oddball paradigm with neutral, oddball, self‐relevant, and cannabis‐related stimuli. Cannabis users exhibited increased amplitude in P3 and faster latencies in the P3, N2, and P2 components in response to self‐relevant stimuli compared to controls during baseline that normalized after rTMS. These results suggest that cannabis users exhibited heightened salience to external self‐relevant stimuli that were modulated after rTMS. PCC dysfunction in cannabis users may be related to abnormalities in processing salient stimuli, such those during cue‐reactivity, and provides a potential target for cannabis use disorder intervention.

## Introduction

The processing of external salient stimuli (i.e., exteroceptive processing) is critical for responding and adapting to the environment. The posterior cingulate cortex (PCC) and precuneus have been implicated in exteroceptive processing (Fransson, 2005; Lou *et al*., 2010; Luber *et al*., 2012) and exhibit activation that is correlated with the default mode network (Fox *et al*., 2005; Raichle, 2011; Utevsky *et al*., 2014). This correlation with the default mode network, a network of brain regions that exhibits activation during resting state and deactivation when engaged in a task, is thought to be critical for continuously monitoring the environment for self‐relevant stimuli. This constant monitoring is imperative to quickly identify and react to relevant external stimuli (e.g., predators) and thus, is likely a readily available attentional resource (Gusnard & Raichle, 2001; Raichle *et al*., 2001; Corbetta *et al*., 2008; Uddin, 2015). Determining the external stimulus’ relevance to the self is also critical in selecting the appropriate response and has been reported to be driven by the PCC (Schilbach *et al*., 2008; Davey *et al*., 2016).

Exteroception is of particular importance in substance abuse as increased awareness to drug‐related cues may underlie increased craving and lead to drug‐seeking behavior (Siegel, 2005; Littel *et al*., 2012; Paulus *et al*., 2013; Cadet *et al*., 2014; DeWitt *et al*., 2015). In substance abuse, neuroimaging studies indicate greater activity in the PCC and precuneus with drug‐cue exposure in cocaine (Grant *et al*., 1996), alcohol (Tapert *et al*., 2003), nicotine (McBride *et al*., 2006; Brody *et al*., 2007; McClernon *et al*., 2009; Claus *et al*., 2013), and cannabis (Feldstein Ewing *et al*., 2012; Filbey & Dunlop, 2014; Filbey *et al*., 2016) users. Specifically in cannabis users, greater activation in the PCC and precuneus in response to cannabis cues indicates a hyper‐sensitization to these cues (Filbey *et al*., 2016) and has also been associated with decreased performance on the Iowa Gambling Task that assesses strategic decision‐making (Bolla *et al*., 2005; Wesley *et al*., 2011). Together with studies reporting increased cue‐elicited craving in cannabis users (Wölfling *et al*., 2008; Filbey *et al*., 2009; Filbey & DeWitt, 2012), these findings suggest that cannabis users not only have increased salience to cannabis cues and cue‐elicited craving, but also compromised decision‐making that may lead to subsequent drug‐seeking behavior.

The link between processing of external self‐relevant stimuli and increased salience to cannabis cues that is purportedly driven by the PCC and precuneus provides an interesting approach to potentially modulate craving via manipulation of these regions in cannabis users. A method for manipulation of these regions is through repetitive transcranial magnetic stimulation (rTMS). rTMS induces neuroplastic changes through the application of magnetic stimuli (produced in a coil of wire called the magnetic coil) directly to a brain area and can modulate cortical excitability using either inhibitory low frequency (≤ 1 Hz; LF) or excitatory high frequency (≥ 5 Hz; HF) stimulation. This non‐invasive neuromodulation technique has been applied to relieve symptoms for neurological and psychiatric disorders (McNamara *et al*., 2001; Lomarev *et al*., 2006; Poulet *et al*., 2010) and to reduce craving in nicotine (Amiaz *et al*., 2009; Hayashi *et al*., 2013; Li *et al*., 2013; Pripfl *et al*., 2014), cocaine (Camprodon *et al*., 2007; Politi *et al*., 2008), and alcohol users (Mishra *et al*., 2010; De Ridder *et al*., 2011; Rapinesi *et al*., 2013). In cannabis users, the literature has been limited with only two studies examining the effects of rTMS. Fitzgerald and colleagues indicated a reduction in cortical inhibition after single and paired rTMS targeting the motor cortex in cannabis users in comparison to control participants (Fitzgerald *et al*., 2009). Sahlem and colleagues reported no change in craving after a single session of 10 Hz rTMS to the left dorsolateral prefrontal cortex (Sahlem *et al*., 2018). While these studies have largely focused on applying rTMS using a figure‐eight coil to the dorsolateral prefrontal cortex (Bellamoli *et al*., 2014; Gorelick *et al*., 2014; Salling & Martinez, 2016), the above evidence suggests that the PCC and precuneus may also be appropriate targets for applying rTMS to modulate response to self‐relevant stimuli and reduce craving. Targeting deeper brain areas, such as the PCC, have shown to be possible using more advanced rTMS coil designs than the figure‐8 coil (Deng *et al*., 2013, 2014); however, only few rTMS studies have investigated the PCC using this double‐cone coil (Hayward *et al*., 2007; Vanneste *et al*., 2012).

Thus, the aim of this study was to modulate response to external self‐relevant stimuli using double‐cone coil rTMS by targeting the PCC and precuneus and to investigate the role of these regions in processing salient stimuli in cannabis users and non‐using controls. To assess neural response to external self‐relevant stimuli, we modified the oddball paradigm to include self‐relevant and cannabis‐related stimuli that occurred infrequently and were expected to elicit the P3 response (Gray *et al*., 2004) as well as the preceding P2 and N2 responses measured through electroencephalography (EEG). We hypothesized a greater P2, N2, and P3 response to self‐relevant stimuli during baseline compared to after HF rTMS (i.e., 10 Hz) in both cannabis users and non‐using controls and no change after LF rTMS (i.e., 1 Hz) as this condition was used as a comparison measure. We also predicted a greater response to cannabis‐related stimuli during baseline compared to after HF rTMS in cannabis users due to the high salience of these cues to users, but not in controls.

## Materials and methods

### Participants

Twenty adult participants were recruited from the general community to take part in this study. Of these, 10 reported having ≥ 7 days of cannabis use in the preceding 30 days (mean age = 27.1 ± 4.5; five females) and 10 participants reported having < 5 separate occasions of cannabis use in their lifetime (mean age = 33.9 ± 14.1; five females; see Table 1 for demographic information). Cannabis use was verified by quantification of delta‐9‐tetrahydrocannabinol (THC) metabolites in urine via gas chromatography/mass spectrometry (GC/MS) from Quest Diagnostics (https://www.questdiagnostics.com). The inclusion criteria for all participants included English proficiency and right‐handedness. All participants provided written informed in accordance with World Medical Association Declaration of Helsinki and the research protocol was approved by the Institutional Review Board (IRB) of the University of Texas at Dallas. The exclusion criteria included any history of brain injury, neurological of psychiatric diagnoses, or any EEG or rTMS contraindications, regular tobacco use and current alcohol dependence as assessed by the Structured Clinical Interview for DSM‐IV (SCID; First *et al*., 2002).

**Table 1:** Participant demographics (mean ± SD)

	Cannabis users	Non‐using controls	*P*‐value
*N*	10	10	–
Age (years)	27.1 ± 4.5	33.9 ± 14.1	0.16
Gender (M/F)	5/5	5/5	1.0
Years of education	13.7 ± 3.1	16.5 ± 1.9	0.024*
Ethnicity			
Hispanic/Latino	2	1	
Non‐Hispanic/Latino	8	9	0.56
Race			
Caucasian	5	5	
African American	3	1	
Asian	0	3	
Other	2	1	1.0
Number of alcohol drinking days in preceding 90 days	12.3 ± 12.8	5.7 ± 8.5	0.077
Number of smoking days in preceding 90 days	17.9 ± 37.7	0	0.15
Number of cannabis use days in preceding 90 days	76.7 ± 18.1	0	< 0.001*
Number of participants meeting criteria for cannabis abuse (current/lifetime)	2/5	0/0	0.12/0.006*
Number of participants meeting criteria for cannabis dependence (current/lifetime)	1/3	0/0	0.28/0.047*
MPS	3.8 ± 4.4	–	–
MCQ	301.0 ± 184.2	12.5 ± 31.4	< 0.001*

*P < 0.050. MCQ, Marijuana Craving Questionnaire; M/F, male/female; MPS, Marijuana Problem Scale.

The participants were instructed to abstain from cannabis use 24 h prior to their sessions to ensure no acute intoxication during data collection. Abstinence was verified via the Time Line Follow Back (Sobell & Sobell, 1992), self‐reported date and time of last use, and absence of behavioral signs of cannabis intoxication.

### Behavioral measures

The TLFB was also used to determine the number of cannabis use days in the 90 days preceding the session. The Marijuana Craving Questionnaire (MCQ; Heishman *et al*., 2009) was used to assess cannabis craving and the Marijuana Problem Scale (MPS; Stephens *et al*., 2002) was used to assess impact of cannabis use on daily life functioning.

### Modified oddball paradigm

Similar to Gray *et al*. (2004), we modified the traditional visual oddball paradigm to include self‐relevant stimuli as well as cannabis‐related stimuli. On each trial, a neutral, oddball, self‐relevant, or cannabis‐related word was displayed on the screen. For all categories of stimuli, half of the stimuli appeared in solid text and half appeared in striped text. Participants were instructed to press the left button if the stimulus appeared in solid text and the right button if the stimulus appeared in striped text.

As described by Gray *et al*. (2004), self‐relevant words for each participant were extracted prior to the experiment. Specifically, all participants completed a questionnaire in order to obtain personal information (i.e., self‐relevant) such as “mother's first name,” “high school,” “pet's name,” and “hometown zip code” (Gray *et al*., 2004) prior to the task. Neutral stimuli were similar information, but irrelevant or unrelated to the participant. This was verified by asking participants to rate whether each neutral stimuli was relevant to them from a scale of 0 (“not at all relevant”) to 4 (“extremely relevant”). Oddball stimuli were neutral stimuli (irrelevant) but appeared in an oddball color, i.e., blue (all other stimuli appeared in black). Cannabis‐related words included “marijuana,” “joint,” “high,” “weed,” and “420.” There were 280 neutral trials (70%), 40 oddball trials (10%), 40 self‐relevant trials (10%), and 40 cannabis‐related trials (10%) for a total of 400 trials.

### Study design

**Figure 1:**
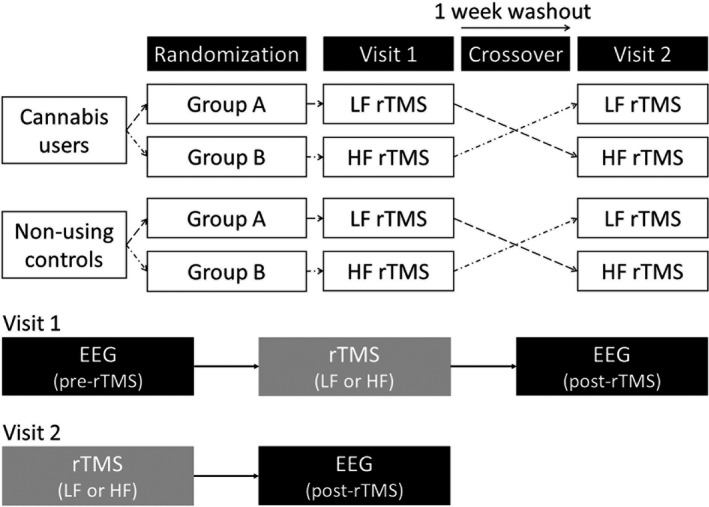
Randomized crossover design of the study. The low and high frequency repetitive transcranial magnetic stimulation sessions were counter‐balanced between the participants. HF, high frequency rTMS (10 Hz); LF, low frequency rTMS (1 Hz).

A randomized crossover study design was implemented with two data collection sessions 1 week apart (Fig. 1). In the first session, participants underwent EEG data collection during the task at a baseline session followed by rTMS administration in either the low frequency (LF; 1 Hz) or high frequency (HF; 10 Hz) condition. Participants repeated the EEG data collection during the modified oddball task after rTMS. In the second session, the participant underwent the LF or HF rTMS administration and the EEG data collection during the task. The LF and HF rTMS sessions were counter‐balanced between the participants. This study design resulted in EEG data under three rTMS conditions: baseline, after LF‐rTMS, and after HF‐rTMS.

### rTMS administration

Repetitive transcranial magnetic stimulation was performed using a Magstim Rapid2 stimulator (Magstim Co. Ltd.) attached to a double‐cone coil (DCC; P/N 9902‐00; Magstim Co. Ltd.) placed over the parietal cortex, 4 cm behind the motor strip (Hayward *et al*., 2007; Vanneste *et al*., 2012), as localized by rTMS when determining the individual motor threshold. We opted to use a DCC with two angled windings over the medial parietal cortex to target the precuneus/PCC as the DCC has shown to be able to reach stimulation targets at depth of 3–4 cm (Kakuda *et al*., 2013; Lu & Ueno, 2017) and has previously been shown to target the PCC through this location (Hayward *et al*., 2007; Vanneste *et al*., 2012). The coil was positioned approximately halfway between the inion and the zenith of the vertex and fixed with a mechanical arm following the procedure of Vanneste and colleagues (Vanneste *et al*., 2012).

Before each rTMS session, the individual motor threshold was first determined by delivering single pulse TMS placing a figure‐eight coil (Double 70 mm Air cooled Coil; Magstim Co. Ltd.) over the motor cortex. The threshold was established by using the criterion of the lowest intensity of stimulation that would result in visually perceptible movements of the participant's left thumb 50% of the time, following stimulation of the finger and thumb area of the primary motor cortex (Kozel *et al*., 2018).

Repetitive transcranial magnetic stimulation consisted of short trains of pulses given at an intensity of 80% of each participant's resting motor threshold (RMT) or at 45% maximum stimulator output (MSO) when 80% of the individual RMT exceeded 45% MSO. This is done because TMS with the DCC at high intensities is very unpleasant for the participants, more so than figure‐eight coil stimulation (De Ridder *et al*., 2011). The participants received repeated stimulation at 1 and 10 Hz in two randomized sessions with a 1‐week intersession interval. The purpose of the 1‐week wash‐out period was to avoid carry over effects (e.g., Schuwerk *et al*., 2014). Each rTMS session consisted of 2000 pulses. The 1 Hz session consisted of 400 trains of five pulses in 5 s with an intertrain interval of 5 s, whereas the 10 Hz session consisted of 40 trains of 50 pulses over 5 s with an intertrain interval of 50 s. The waiting time between the trains of pulses varied between the 1 and the 10 Hz session to reach more matching stimulation time and to avoid potential overheating of the coil, as the DCC does not have a built‐in cooling system. The participants wore earplugs during the rTMS session.

### EEG data acquisition and analysis

Electroencephalography data were recorded from a Neuroscan Quickcap with 64 electrodes, Neuroscan Synamps2 amplifier, and Scan 4.3.2 software. The electrodes were preconfigured in the EEG net according to the international 10–20 system and placed with reference to the nasion and inion. The reference electrode was placed on the left mastoid. Data were re‐referenced offline with respect to both left and right mastoids. Electrode impedances were maintained below 7 kΩ. The data were recorded at a sampling frequency of 1000 Hz. EEG was recorded during the modified oddball paradigm.

Offline preprocessing of the EEG data was conducted in EEGLAB (Delorme & Makeig, 2004) and additional analyses were conducted in matlab (MathWorks, Natick, MA) using custom scripts. EEG signals were band‐pass filtered at 0.1–55 Hz and eye artifacts were removed using independent component analysis.

The EEG data were segmented 200 ms prior to stimulus onset and 1000 ms after stimulus onset for each trial. Each stimulus condition was averaged individually for nine electrode sites (F3, Fz, F4, C3, Cz, C4, P3, Pz, and P4) for each participant. The P2 (150–200 ms after stimulus onset), N2 (250–300 ms after stimulus onset), and P3 (300–600 ms after stimulus onset) components were analyzed in response to the stimuli as these are characteristic event‐related potentials (ERPs) elicited in the standard oddball paradigm.

### Statistical analyses

A five‐way mixed factorial (2 × 2 × 3 × 3 × 3) anova with Group (cannabis user, non‐using control) as the between subjects variable and rTMS Condition (baseline, LF rTMS), Stimulus (oddball, self‐relevant, cannabis‐related), Region (frontal, central, parietal), and Laterality (left, midline, right) as the within subject variables was conducted separately for the P3 component amplitude and latency. A separate five‐way mixed factorial anova was conducted to compare baseline and HF rTMS. Greenhouse‐Geisser corrected *P*‐values were used when sphericity was violated. Significance was defined at *P *<* *0.050. *Post hoc* analyses were conducted on significant effects as well as on pairwise and between‐group differences determined *a priori* using a Bonferroni correction. Similar separate five‐way mixed factorial anovas were conducted for the P2 and N2 components. To assess the effect of rTMS on craving, a repeated measures anova was conducted on the MCQ scores recorded during baseline, after LF rTMS, and after HF rTMS in cannabis users. The amplitude and latency of the three components was also correlated with measures of cannabis use (SCID symptom count, MPS score, and number of cannabis use days in the preceding 90 days) and subacute effects of cannabinoids (hours since last use and THC metabolite levels) in the cannabis using group.

## Results

### Participants

There were no significant differences in age, gender, or ethnicity between the cannabis users and non‐using controls; although, the controls as a group were older compared to the users. While there were no significant differences between number of alcohol drinking and smoking days in the preceding 90 days, on average the user group drank and smoked on more days (mean alcohol drinking days = 12.3 ± 12.8; mean smoking days = 17.9 ± 37.7) compared to the control group (mean alcohol drinking days = 5.7 ± 8.5; mean smoking days = 0). The user group had significantly greater cannabis use compared to controls in the preceding 90 days (cannabis users mean = 76.7 ± 18.1 days; controls mean = 0; *P *<* *0.001). Two of the cannabis users met criteria for current cannabis abuse, five met criteria for lifetime cannabis abuse, one met criteria for current cannabis dependence, and three met criteria for lifetime cannabis dependence.

### Behavioral measures

The repeated measures anova found no differences in MCQ scores during baseline, after LF rTMS, and after HF rTMS (*F*_2,18_ = 1.6, *P *=* *0.22).

### P2 component

#### LF rTMS

The mixed factorial anova found a significant main effect of Region (*F*_1.3,22.5_ = 7.3, *P* = 0.009, partial η^2^ = 0.29) and a Region x Laterality interaction (*F*_2.8,50.3_ = 6.4, *P *=* *0.001, partial η^2^ = 0.26) in P2 amplitude. *Post hoc* analyses found increased amplitude in the frontal electrodes compared to the central electrodes on the left (*P *=* *0.021, Cohen's *d* = 0.34), midline (*P* = 0.001, Cohen's *d* = 0.41), and right (*P* < 0.001, Cohen's *d* = 0.53) hemispheres. Mixed factorial anovas on the latency of the P2 component indicated a main effect of Region (*F*_2,36_ = 19.0, *P *<* *0.001, partial η^2^ = 0.51) and Group (*F*_1,18_ = 5.5, *P *=* *0.030, partial η^2^ = 0.24). There were also significant interactions between Laterality × Stimulus (*F*_4,72_ = 4.2, *P *=* *0.004, partial η^2^ = 0.19), Laterality × Stimulus × Group (*F*_4,72_ = 2.8, *P* = 0.034, partial η^2^ = 0.13), and rTMS Condition × Region × Laterality × Stimulus (*F*_8,144_ = 2.2, *P* = 0.030, partial η^2^ = 0.11). *Post hoc* analyses found slower response in controls compared to users overall and faster response in the left and right frontal and central regions compared to the parietal regions in response to self‐relevant stimuli (left frontal, *P *=* *0.009, Cohen's *d* = 1.0; right frontal, *P *<* *0.001, Cohen's *d* = 1.2; right central, *P *=* *0.020, Cohen's *d* = 0.76) and right hemisphere in response to cannabis‐related stimuli (frontal, *P *=* *0.002, Cohen's *d* = 1.3; central, *P *=* *0.002, Cohen's *d* = 1.1) during baseline. These differences remained after LF rTMS in response to self‐relevant stimuli (left frontal, *P *=* *0.006, Cohen's *d* = 1.1; midline frontal, *P *=* *0.009, Cohen's *d* = 1.0; right frontal, *P *=* *0.001, Cohen's *d* = 1.4) and cannabis‐related stimuli (right frontal, *P *=* *0.005, Cohen's *d* = 1.2; right central, *P *=* *0.029, Cohen's *d* = 0.65).

#### HF rTMS

The mixed factorial anova on P2 amplitude found a main effect of Region (*F*_1.2,20.8_ = 5.6, *P* = 0.007, partial η^2^ = 0.24) and a Region x Laterality interaction (*F*_4,72_ = 3.3, *P* = 0.015, *P* = 0.16). *Post hoc* analyses found increased amplitude in the frontal region compared to the central region in the left (*P *=* *0.046, Cohen's *d* = 0.28), midline (*P *=* *0.005, Cohen's *d* = 0.33), and right (*P *<* *0.001 Cohen's *d* = 0.46) hemispheres. Mixed factorial anova on P2 latency found a main effect of Region (*F*_2,36_ = 21.6, *P *<* *0.001, partial η^2^ = 0.55) and Laterality (*F*_2,36_ = 6.4, *P *=* *0.004, partial η^2^ = 0.26). There were also interaction effects between Region × Laterality (*F*_2.8,50.1_ = 3.2, *P *=* *0.033, partial η^2^ = 0.15) and Group × Stimulus × Laterality (*F*_4,72_ = 3.1, *P *=* *0.021, partial η^2^ = 0.15). The *post hoc* analyses revealed no significant differences.

**Figure 2:**
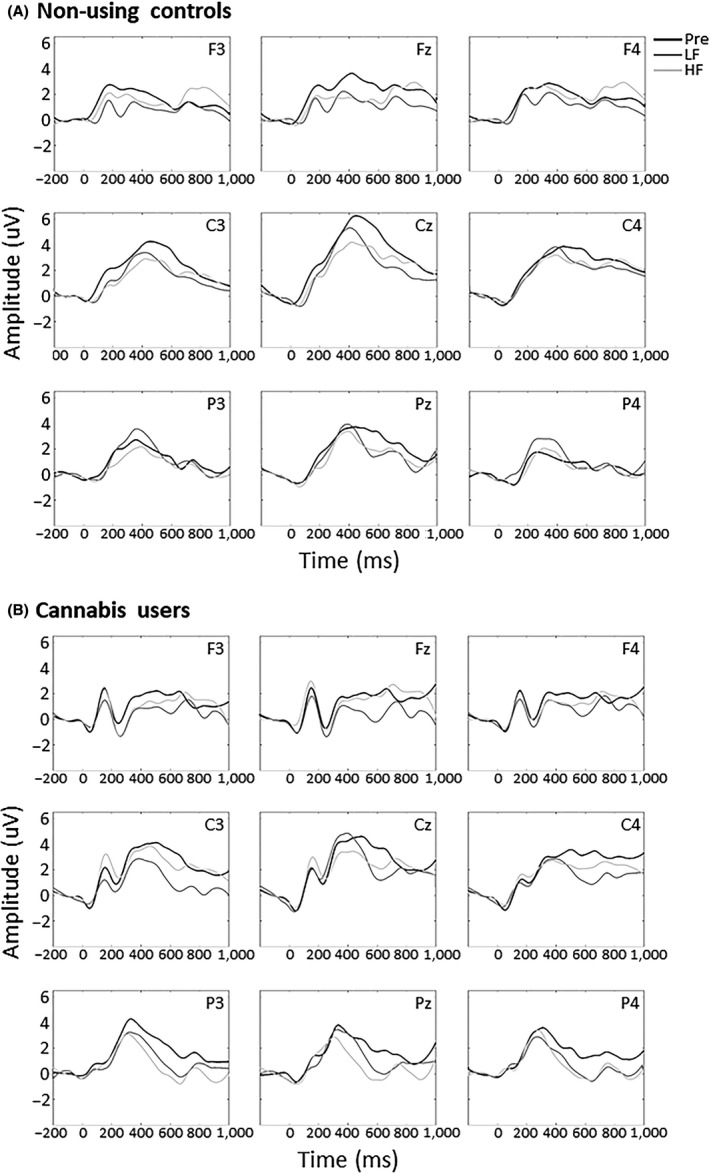
(A) Amplitude and (B) latency of the P2 component in response to self‐relevant stimuli in the nine electrodes of interest. Cannabis users exhibited an increased P2 amplitude in P4 (*P* = 0.032) and faster response in frontal and central electrodes during baseline (both Fz and F4, *P* = 0.011; Cz, *P* = 0.035) and after LF rTMS (F3, *P* = 0.042; Fz, *P* = 0.036; F4, *P* = 0.011; C3, *P* = 0.039) compared to non‐using controls. There were no differences between the groups after HF rTMS, except in F4 (*P* = 0.045). Error bars indicate standard error, *P < 0.05, ^+^ *P* < 0.08, Pre = baseline. HF, high frequency rTMS (10 Hz); LF, low frequency rTMS (1 Hz).

**Figure 3:**
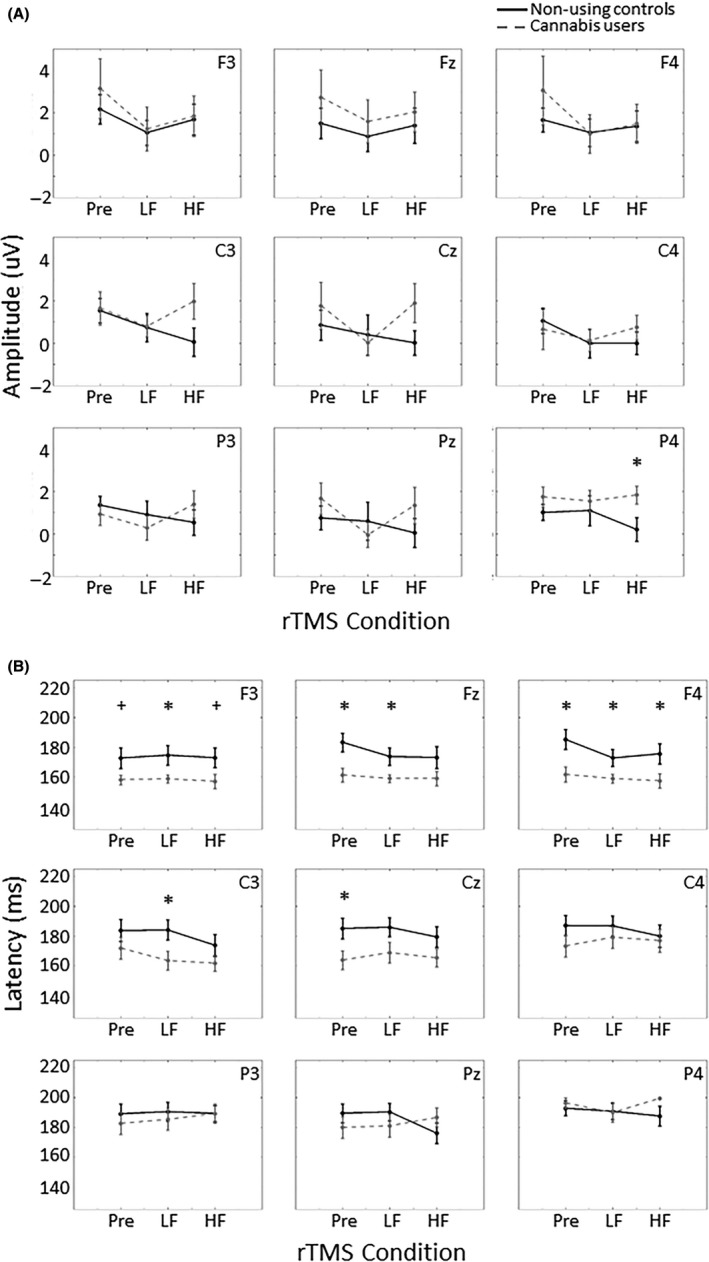
Averaged event‐related potential waveform of self‐relevant stimuli of (A) non‐using controls and (B) cannabis users in the nine electrodes of interest, Pre = baseline. HF, high frequency rTMS (10 Hz); LF, low frequency rTMS (1 Hz).

The *a priori* determined *t* tests analysis found a significantly higher amplitude in response to self‐relevant stimuli in cannabis users compared to controls in the P4 electrode (*P *=* *0.032, Cohen's *d* = 1.04; see Fig. 2A). There was also a faster P2 response in cannabis users after LF rTMS in the F3 electrode (*P* = 0.042, Cohen's *d* = 0.98) and approached significance during baseline (*P* = 0.071, Cohen's *d* = 0.86) and after HF rTMS (*P* = 0.070, Cohen's *d* = 0.86; see Fig. 2B). In the Fz electrode, cannabis users exhibited faster latency during baseline (*P* = 0.011, Cohen's *d* = 1.26) and after LF rTMS (*P* = 0.036, Cohen's *d* = 1.01), but not after HF rTMS (*P* = 0.13, Cohen's *d* = 0.71). Cannabis users also exhibited faster latency throughout the rTMS conditions in the F4 electrode (baseline, *P* = 0.011, Cohen's *d* = 1.27; LF rTMS, *P* = 0.046, Cohen's *d* = 0.96; HF rTMS, *P* = 0.045, Cohen's *d* = 0.97). In the C3 electrode, cannabis users exhibited faster latency after LF rTMS (*P* = 0.039, Cohen's *d* = 1.00), but not during baseline (*P* = 0.27, Cohen's *d* = 0.51) or after HF rTMS (*P* = 0.21, Cohen's *d* = 0.59) and only during baseline in the Cz electrode (*P* = 0.035, Cohen's *d* = 1.10). The averaged ERP response to self‐relevant stimuli is depicted in Fig. 3. There were no differences in P2 response to cannabis‐related stimuli between the groups.

### N2 component

#### LF rTMS

The mixed factorial ANOVA on the amplitude of the N2 component indicated a main effect of Region (*F*_1.3,23.6_ = 6.8, *P *=* *0.010, partial η^2^ = 0.27) and a significant interaction between Region × Laterality (*F*_4,72_ = 5.4, *P *=* *0.001, partial η^2^ = 0.23). *Post hoc* analysis revealed increased N2 amplitude in the parietal region compared to the frontal region in both the left (*P *=* *0.017, Cohen's *d* = 0.77) and right (*P *=* *0.024, Cohen's *d* = 0.73) hemispheres. For N2 latency, there was a main effect of Laterality (*F*_2,36_ = 7.1, *P *=* *0.003, partial η^2^ = 0.28) and a significant interaction between Region × Laterality (*F*_2.7,48.9_ = 6.1, *P *=* *0.002, partial η^2^ = 0.25). *Post hoc* analyses did not reveal significant differences.

#### HF rTMS

The mixed factorial anova on N2 amplitude indicated a main effect of Region (*F*_1.3,22.8_ = 5.7, *P *=* *0.020, partial η^2^ = 0.24) and Laterality (*F*_2,36_ = 4.5, *P *=* *0.018, partial η^2^ = 0.20), and a significant interaction between rTMS Condition × Region × Stimulus × Group (*F*_2.4,43.8_ = 3.4, *P *=* *0.034, partial η^2^ = 0.16). *Post hoc* analysis revealed increased amplitude in the right hemisphere compared to the midline (*P *=* *0.021, Cohen's *d* = 0.21). Cannabis users also exhibited increased amplitude in the parietal region compared to the frontal region after HF rTMS in response to the self‐relevant stimuli (*P *=* *0.011, Cohen's *d* = 1.3) and the cannabis‐related stimuli (*P *=* *0.048, Cohen's *d* = 0.84). For N2 latency, there was a main effect of Laterality (*F*_2,36_ = 5.8, *P *=* *0.006, partial η^2^ = 0.25) and interaction effects between Group x Laterality (*F*_2,36_ = 4.6, *P *=* *0.017, partial η^2^ = 0.20), Region × Laterality (*F*_4,72_ = 5.5, *P *=* *0.001, partial η^2^ = 0.23), and rTMS Condition x Stimulus (*F*_4,72_ = 3.4, *P *=* *0.043, partial η^2^ = 0.16). *Post hoc* analyses indicated faster N2 response in users compared to controls in the left hemisphere (*P *=* *0.011, Cohen's *d* = 0.60).

**Figure 4:**
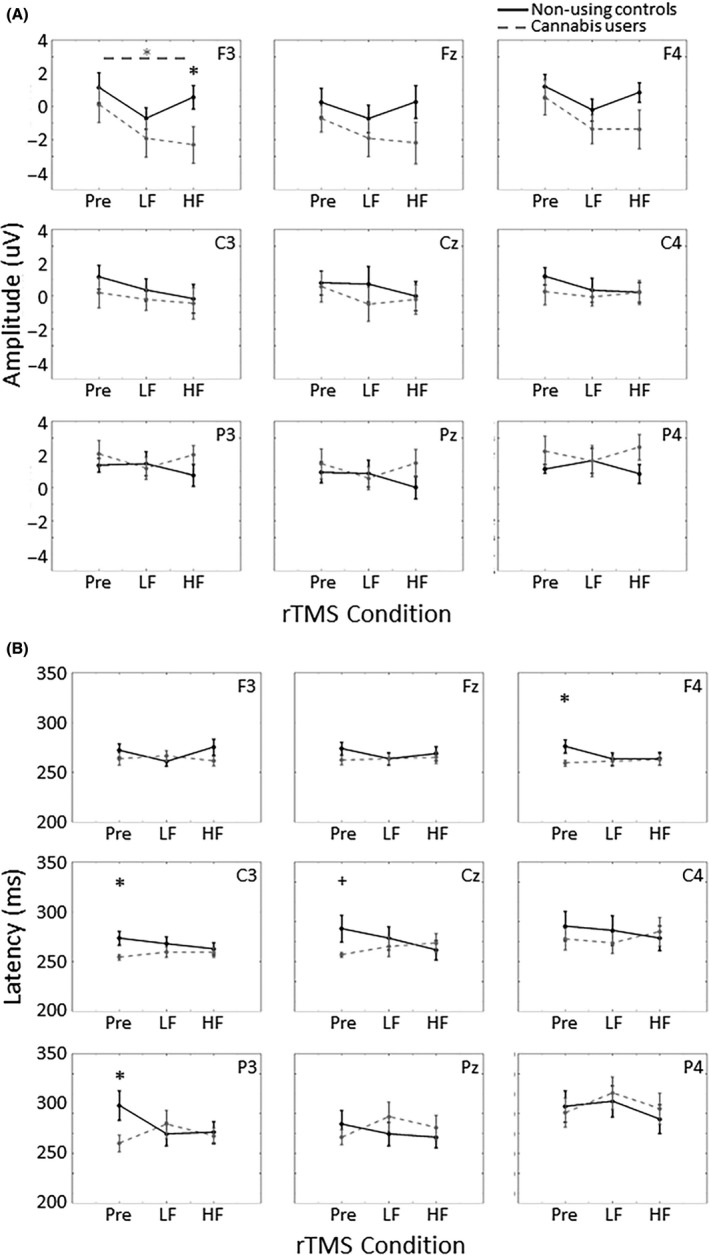
(A) Amplitude and (B) latency of the N2 component in response to self‐relevant stimuli in the nine electrodes of interest. Cannabis users exhibited a reduced N2 amplitude in F3 after HF rTMS compared to non‐using controls (*P* = 0.042) and compared to baseline (*P* = 0.049). Users also exhibited faster N2 response compared to controls during baseline (F4, *P* = 0.037; C3, *P* = 0.021; P3, *P* = 0.039), but not after LF or HF rTMS. Error bars indicate standard error, *P < 0.05, ^+^ *P* < 0.08, Pre = baseline. HF, high frequency rTMS (10 Hz); LF, low frequency rTMS (1 Hz).

A *priori* determined *t* tests found a significant decrease in N2 amplitude after HF rTMS compared to baseline in cannabis users (*P *=* *0.049, Cohen's *d* = 0.70) in the F3 electrode. Cannabis users also exhibited significantly reduced N2 amplitude compared to controls after HF rTMS (*P *=* *0.042, Cohen's *d* = 0.98), but not after LF rTMS (*P *=* *0.36, Cohen's *d* = 0.42) or during baseline (*P *=* *0.47, Cohen's *d* = 0.33). Similar trends were found in the Fz and F4 electrodes, but were not statistically significant (Fig. 4A). There were also differences in latency of N2 response to self‐relevant stimuli (Fig. 4B). Cannabis users exhibited significantly faster N2 response to self‐relevant stimuli in the F4 (*P *=* *0.037, Cohen's *d* = 1.0), C3 (*P *=* *0.021, Cohen's *d* = 1.1) and P3 (*P *=* *0.039, Cohen's *d* = 0.99) electrodes, and approached significance in the Cz electrode during baseline (*P *=* *0.072, Cohen's *d* = 0.85), but not after LF or HF rTMS.

### P3 component

#### LF rTMS

Mixed factor anova on amplitude of the P3 component indicated a main effect of Region (*F*_2,36_ = 6.2, *P *=* *0.005, partial η^2^ = 0.26) and Laterality (*F*_2,36_ = 10.7, *P *<* *0.001, partial η^2^ = 0.37) as well as significant interactions between Region × Laterality (*F*_4,72_ = 7.2, *P *<* *0.001, partial η^2^ = 0.29) and rTMS Condition × Stimulus (*F*_4,72_ = 7.2, *P *=* *0.002, partial η^2^ = 0.29). *Post hoc* analyses indicated increased P3 amplitude in the central region compared to the frontal region in the midline (*P *<* *0.001, Cohen's *d* = 0.74) and left (*P *=* *0.002, Cohen's *d* = 0.55) hemispheres. P3 amplitude was also greater in response to the oddball stimuli compared to the self‐relevant stimuli (*P *=* *0.024, Cohen's *d* = 0.41) and the cannabis‐related stimuli (*P* = 0.005, Cohen's *d* = 0.40) after LF rTMS. For P3 latency, there was a main effect of Region (*F*_1.5,27.6_ = 5.8, *P *=* *0.007, partial η^2^ = 0.24) and Laterality (*F*_1.5,27.6_ = 8.9, *P *=* *0.001, partial η^2^ = 0.33). There were also interaction effects between rTMS Condition × Region (*F*_1.5,26.4_ = 4.0, *P* = 0.043, partial η^2^ = 0.18), Region × Laterality (*F*_4,72_ = 3.5, *P *=* *0.012, partial η^2^ = 0.16), and rTMS Condition × Stimulus (*F*_4,72_ = 5.3, *P* = 0.009, partial η^2^ = 0.23). *Post hoc* analyses indicated slower P3 response during baseline compared to after LF rTMS in the central (*P* = 0.050, Cohen's *d* = 0.40) and parietal (*P* = 0.043, Cohen's *d* = 0.31) regions. Additionally, there was slower P3 response during baseline compared to after LF rTMS in response to the self‐relevant stimuli (*P* = 0.004, Cohen's *d* = 0.45) and slower response to oddball stimuli compared to self‐relevant stimuli after LF rTMS (*P* = 0.004, Cohen's *d* = 0.25).

#### HF rTMS

The mixed factorial anova on amplitude revealed a main effect of Laterality (*F*_1.4,26.1_ = 7.7, *P *=* *0.002, partial η^2^ = 0.30) and significant interactions between Region × Laterality (*F*_2.7,49.0_ = 4.0, *P *=* *0.005, partial η^2^ = 0.18) and rTMS Condition × Laterality × Stimulus (*F*_2.3,41.4_ = 3.2, *P *=* *0.046, partial η^2^ = 0.15). *Post hoc* analyses revealed increase P3 amplitude in the central region compared to the frontal region in the midline (*P *=* *0.007, Cohen's *d* = 0.49) and left (*P *=* *0.017, Cohen's *d* = 0.37) hemispheres. The P3 amplitude was also greater during baseline compared to after HF rTMS in response to self‐relevant stimuli in the left (*P *=* *0.012, Cohen's *d* = 0.54), midline (*P *=* *0.009, Cohen's *d* = 0.62), and right (*P *=* *0.037, Cohen's *d* = 0.49) hemispheres. For P3 latency, there was a main effect of Region (*F*_1.5,26.5_ = 5.2, *P *=* *0.010, partial η^2^ = 0.23), Laterality (*F*_2,36_ = 7.1, *P *=* *0.003, partial η^2^ = 0.28), and Stimulus (*F*_2,36_ = 5.3, *P* = 0.009, partial η^2^ = 0.23). There were also significant interactions between rTMS Condition × Stimulus (*F*_2,36_ = 5.8, *P* = 0.007, partial η^2^ = 0.24), rTMS Condition × Region × Laterality (*F*_4,72_ = 3.2, *P* = 0.018, partial η^2^ = 0.15), and rTMS Condition × Region × Stimulus (*F*_4,72_ = 2.6, *P* = 0.041, partial η^2^ = 0.13). *Post hoc* analyses revealed slower P3 response to oddball stimuli after HF rTMS compared to baseline in the frontal region (*P *=* *0.030, Cohen's *d* = 0.57), but faster response to self‐relevant stimuli after HF rTMS compared to baseline in the central region (*P *=* *0.011, Cohen's *d* = 0.59).

**Figure 5:**
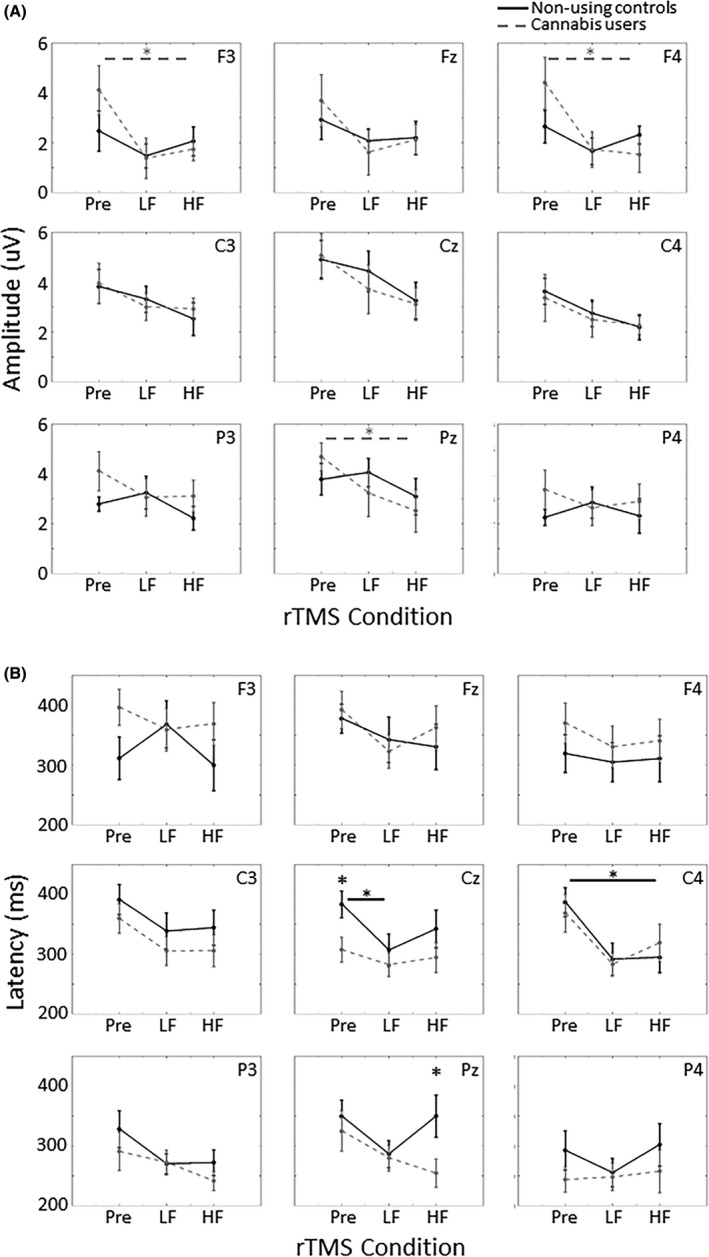
(A) Amplitude and (B) latency of the P3 component in response to self‐relevant stimuli in the nine electrodes of interest. Cannabis users exhibited a decreased P3 amplitude after HF rTMS compared to baseline in F3 (*P* = 0.021), F4 (*P* = 0.005), and Pz (*P* = 0.008). Cannabis users also exhibited faster P3 response compared to non‐using controls during baseline in Cz (*P* = 0.022) and after HF rTMS in Pz (*P* = 0.037). Error bars indicate standard error, *P < 0.05, ^+^ *P* < 0.08, Pre = baseline. HF, high frequency rTMS (10 Hz); LF, low frequency rTMS (1 Hz).

The *t* tests determined *a priori* revealed significantly greater P3 amplitude in response to self‐relevant stimuli during baseline compared to after HF rTMS in the F3 (*P *=* *0.021, Cohen's *d* = 0.98), F4 (*P *=* *0.005, Cohen's *d* = 1.0), and Pz (*P *=* *0.008, Cohen's *d* = 0.95) electrodes in cannabis users (Fig. 5A). Unlike the amplitude, differences in P3 latency in response to self‐relevant stimuli were driven by differences in the control group with slower latency during baseline compared to after HF rTMS in the C4 electrode (*P *=* *0.041, Cohen's *d* = 1.2) and after LF rTMS in the Cz electrodes (*P *=* *0.004, Cohen's *d* = 0.98). There were also group differences in P3 latency with cannabis users exhibiting faster latency compared to controls during baseline in Cz (*P *=* *0.022, Cohen's *d* = 1.1) and after HF rTMS in Pz (*P *=* *0.037, Cohen's *d* = 1.0).

### Correlation with cannabis use measures

There were no significant correlations between the cannabis use measures and the latency and amplitude of the three components.

## Discussion

The aim of this study was to examine the role of the PCC and precuneus in processing external self‐relevant stimuli in cannabis users and non‐using controls. We used rTMS to modulate activity in these regions and assessed response to self‐relevant and cannabis‐related stimuli by recording ERPs. We found that cannabis users exhibited an increased response to self‐relevant stimuli compared to controls during baseline that normalized after HF rTMS to the PCC and precuneus.

### Cannabis users exhibited heightened exteroceptive processes

The P3 is elicited in response to novel stimuli and has been shown to also be evoked by self‐relevant stimuli (Gray *et al*., 2004). Cannabis users exhibited increased response to self‐relevant stimuli during baseline that was reduced after HF rTMS, suggested a decrease in response to externally salient stimuli after rTMS. Users also exhibited faster P3 response latency to self‐relevant stimuli that did not change after rTMS. Controls, however, exhibited faster P3 latency after LF rTMS in Cz and HF rTMS in C4 compared to baseline, suggesting faster processing of the stimuli (Folstein & Van Petten, 2007). The increased amplitude and faster latency of the P3 response in cannabis users compared to controls suggests increased exteroceptive salience in users not just for cannabis cues (Filbey *et al*., 2009, 2016; Feldstein Ewing *et al*., 2012; Filbey & Dunlop, 2014; DeWitt *et al*., 2015), but any external self‐relevant stimuli. The group difference present during baseline in Cz indicates that controls exhibited slower latency compared to cannabis users, but no differences were found after LF and HF rTMS. In contrast is the finding that users exhibited reduced amplitude after HF rTMS compared to baseline. Together, these findings suggest that rTMS may have a modulatory effect on both latency and amplitude. While the exact nature of the modulation remains unclear, this further indicates that rTMS to the PCC and precuneus may modulate exteroceptive processes. The PCC and precuneus are hubs in the default mode network and involved in determining relevant stimuli from the environment and responding appropriately (Schilbach *et al*., 2008; Davey *et al*., 2016).

The N2 response often precedes the P3 response to infrequent stimuli in the oddball paradigm (Naatanen & Picton, 1986; Luck & Hillyard, 1994) and is thought to reflect cognitive control and response inhibition (Schmajuk *et al*., 2006; Folstein & Van Petten, 2007). Similar to the P3 component, cannabis users exhibited faster N2 latency during baseline that normalized after LF and HF rTMS. Studies have previously reported that cannabis users exhibit greater activity to inhibit an ongoing response in the stop signal task (Filbey & Yezhuvath, 2013), indicating impaired response inhibition. This deficient inhibitory control may be reflected in a faster N2 response that does not allow for the exertion of inhibition. Future studies may examine whether this change in N2 is associated with an increase in response inhibition that may further play a role in decreasing craving after rTMS.

The P2 response is also enhanced in response to infrequent stimuli and precedes the N2 response (Luck & Hillyard, 1994). While it is unclear what cognitive processes it reflects (Crowley & Colrain, 2004), some studies suggest that the P2 component may respond to stimuli that are associated with threat (Bar‐Haim *et al*., 2005). Cannabis users exhibited faster latency that persisted after rTMS. This increased latency is consistent with the P3 and N2 components and further suggests faster processing of external self‐relevant stimuli in users. This is supported by recent findings that there is increased cortical activity during resting state in cannabis users (Prashad *et al*., 2018) that may be related to the enhanced neural response to self‐relevant stimuli. These results also indicate that rTMS may not have an effect on the early sensory processing reflected in the P2.

### Cannabis users did not exhibit increased salience to cannabis‐related stimuli

We predicted a greater response to cannabis‐related stimuli in cannabis users because of their inherent salience to cannabis cues; however, there were few differences between groups and rTMS conditions in response to cannabis‐related stimuli. This lack of difference in response to cannabis cues is inconsistent with the current literature, but may reflect a requirement of additional rTMS sessions to effect change as well as the sample of users included in this study. While the cannabis users were almost daily users (76.7 ± 18.1 days of cannabis use in the preceding 90 days), most did not meet the criteria for abuse or dependence (current or lifetime; see Table 1). Most studies examining response to cannabis cues have included samples of daily heavy users (Singleton *et al*., 2002; Haughey *et al*., 2008; Wölfling *et al*., 2008; McRae‐Clark *et al*., 2011; Filbey *et al*., 2016) and have found differences in response between SCID‐IV dependent and non‐dependent cannabis users (Filbey & Dunlop, 2014). These differences may also exist in heavy and light users such that light users are similar to non‐using controls as found in previous studies (Pope *et al*., 2001; Bolla *et al*., 2002) and should be further explored.

### Effect of rTMS on craving

No differences were found between MCQ during baseline, after LF rTMS, and after HF rTMS. While neuromodulation studies in nicotine, alcohol, and cocaine users (Bellamoli *et al*., 2014; Gorelick *et al*., 2014; Salling & Martinez, 2016; Coles *et al*., 2018) have found decreases in craving after rTMS, there is only one other study examining the effect of rTMS (targeting the dorsolateral prefrontal cortex) in cannabis users and reported no change in craving after one session (Sahlem *et al*., 2018). It may be that subsequent rTMS are necessary to modulate craving as was suggested by a study in cocaine users that reported a gradual reduction in craving after daily rTMS sessions (Politi *et al*., 2008).

### Limitations and future directions

The results of the present study are limited in their interpretability due to the small sample sizes in both groups. In addition, some results may reflect the differences between the groups in education, wider age distribution in the non‐using control sample, and increased alcohol use in the cannabis‐using group. A larger sample and tighter distribution may reveal additional differences in neural response. Several trends failed to achieve significance, but should be further investigated in a larger sample size. Of note, the ERP response displays clear positive (P2) and negative (N2) peaks preceding the P3 in cannabis users that may reflect a fast initial processing of the self‐relevant and cannabis‐related stimuli that are absent in controls. While these results did not reach significance, they may be indicative of differences between the groups that the current sample did not have sufficient power to detect. Thus, it is imperative to replicate and extend these findings in a larger sample that is well matched in variables unrelated to cannabis use (e.g., age, education, use of alcohol and nicotine). There were no significant correlations between the amplitude and latency of the three components with measures of cannabis use, suggesting potential limitations of these measures in indicating subacute effects. It is unclear whether the results are a result of subacute effects of cannabinoids, the 24‐h abstinence period, or ongoing cannabis use and may be elucidated in future studies. Nevertheless, these findings represent an important beginning of a research area with limited studies and an approach that has not yet been explored. An inherent limitation in the study design used here is the absence of a sham rTMS condition. Both rTMS sessions provided stimulation to participants at different frequencies (i.e., 1 and 10 Hz), producing a need for sham‐controlled studies. Studies have shown that the traditional sham condition does not sufficiently control for effects of rTMS as participants are not entirely blind to the presence of active or sham rTMS (Loo *et al*., 2000; Duecker & Sack, 2013).

## Conclusions

This study has demonstrated that cannabis users exhibited heightened exteroceptive processes that may reflect the increased activity of the PCC and precuneus previously associated with response to drug cue‐reactivity paradigms in substance using populations. The increased response to external self‐relevant stimuli was modulated by rTMS targeting these regions such that response to self‐relevant stimuli was normalized after rTMS. This modulation further implicates the PCC and precuneus in exteroception and suggests that the increased salience to external self‐relevant stimuli may be reduced through manipulation of exteroceptive processes in cannabis users by targeting these regions.

## Conflict of interest

The authors declare no conflicts of interest.

## Author contributions

All authors designed the experiment, ESD and WTT conducted the experiment, SP analyzed the data and wrote the manuscript, and all authors provided feedback on the manuscript.

**Abbreviations**
DCC: double‐cone coil
EEG: electroencephalography
ERP: event‐related potential
HF: high frequency
LF: low frequency
MCQ: Marijuana Craving Questionnaire
MPS: Marijuana Problem Scale
MSO: maximum stimulator output
PCC: posterior cingulate cortex
RMT: resting motor threshold
rTMS: repetitive transcranial magnetic stimulation
SCID: Structured Clinical Interview for Diagnostic and Statistical Manual of Mental Disorders‐IV
THC: delta‐9‐tetrahydrocannabinol

## Data Availability

The data are available upon request to Francesca Filbey.
