# Supplementary material for: Testing the role of the posterior cingulate cortex in processing salient stimuli in cannabis users: an rTMS study
Source: Eur J Neurosci. 2018 Oct 27;50(3):2357–69. doi: 10.1111/ejn.14194 (PMC6767056; doi:10.1111/ejn.14194)
Supplement: Supplementary file 1 [file EJN-50-2357-s001.docx]

Testing the role of the posterior cingulate cortex in processing salient stimuli in cannabis users: an rTMS study

Filbey, Francesca (proxy) (contact); Prashad, Shikha; Dedrick, Elizabeth; To, Wing Ting; Vanneste, Sven

_____________________________________________________________________________________

| Review timeline: | Submission date: 21/03/2018  Editorial Decision: Major Revision  Revision Received: 02/08/2018  Editorial Decision: Minor Revision  Revision Received: 11/09/2018  Accepted: 17/09/2018 |  |
| --- | --- | --- |
|  |  |  |

Editor: Rita Goldstein

Reviewer 1: Gregory Sahlem

Reviewer 2: Martin Tik

_____________________________________________________________________________________

1st Editorial Decision	09/05/2018

_____________________________________________________________________________________

Testing the role of the posterior cingulate cortex in processing salient stimuli in cannabis users: an rTMS study 

Dear Dr. Filbey, 

Your manuscript has been reviewed by two external reviewers as well as by the Section Editor,  Dr. Rita Goldstein, and ourselves.  The reviews collectively indicate that your experiments generated new and important information.  However, there are several issues that need to be clarified/resolved in a revised version before we can consider it further for publication in the Special Issue of EJN. 

As you can see, both reviewers consider this to be an important and interesting study that fits well with the scope of the Special Issue on Addiction.  They both raise a series of points that need to be addressed.  Probably the most important of these is the low 'n' and the variability that it has introduced.  This raises questions about the what may underlie the differences observed and the validity of the conclusions.  Please address each of the points that they raise in the revised version. 

We also note the following issues that need to be addressed in the revised version. 
- References are not quite in EJN style (too many authors named in in-text citations) and all authors of cited papers should be included in the reference list. 
- The abbreviations list appears to be empty. 
- Higher resolution figures will be needed for publication. 
- Fig 2 and Table 1 are not cited in the text. 

When revising the manuscript, please embolden or underline major changes to the text so they are easily identifiable and DO NOT leave 'track change' formatting marks in your paper.  Please ensure that you provide a text and a figure file for the Graphical Abstract (as detailed in the instructions below).  When carrying out your revisions please refer to the checklist below and visit the EJN author guidelines at www.ejneuroscience.org 

When finalized, please upload your complete revised manuscript onto the website, as a Word file (.doc, or .docx).  Please also ensure that a complete set of tables and figures is included as separate files, even if these have not changed from the originals.  At this stage it is necessary to provide high resolution figures.  Please see important instructions below. 

Please go into https://mc.manuscriptcentral.com/ejn - Author Centre - manuscripts with decisions where you will find a 'create a revision' link under 'actions'. We ask that you please indicate the way in which you have responded to the points raised by the Editors and Reviewers in a letter. Please upload this response letter as a separate Word (.doc or PDF) file using the file designation "Authors' Response to Reviewers" when uploading your manuscript files.  Please DO NOT submit your revised manuscript as a new one.  Also, please note that only the  Author who submitted the original version of the manuscript should submit a revised version. 

If you are able to respond fully to the points raised, we would be pleased to receive a revision of your paper within 12 weeks. 

Thank you for your support of this Special Issue of EJN. 

Best wishes, 

Paul Bolam & John Foxe 
co-Editors in Chief, EJN 


Reviews: 

Reviewer: 1 

Comments to the Author 
Review for EJN-2018-03-25434(ADX) 
Testing the role of the posterior cingulate cortex in processing salient stimuli in cannabis users: an rTMS study 
In this study the authors reasoned that cannabis users have abnormal salience processing to external stimuli, with a particularly abnormal response to cannabis cues. They further reasoned that the area related to salience processing included the PCC/Precuneus, and that stimulation with rTMS may effect salience processing. They subsequently used an EEG paradigm to record ERP’s during neutral, self-relevant, cannabis, and oddball cues, and attempted to use rTMS applied to the vicinity of the PCC/Precuneus with a double cone coil. They found in fact that there were differences in the ERP’s between users and non-users in self-relevant stimuli, that were altered by rTMS stimulation. 
Though this appears to be a well-designed and executed study, and is a well-written manuscript, I believe the authors should consider revising some sections for clarity (see my comments below). Additionally, I believe they should further explore the possibility that some of their findings might be explained by between group differences unrelated to cannabis (age, education, etc.), and also sub-acute effects of cannabis use (it is unclear how long the last cannabis smoking session was prior to the experiment, and how heavy cannabis use was). I think these alternate explanations are of particular importance given there were no between group differences in ERP’s related to cannabis cues. 
All that said, this appears to be an important early study with which to build future research, and future designs may be more highly controlled. 
rTMS administration: 
-Please consider noting why you chose 4cm behind the motor strip. Is this a validated target? If not, how do you know you in fact hit precuneus/PCC? 
-Please consider revising this section for clarity: “Participants received repeated stimulation in random order at 1Hz and 10 Hz, each stimulation session consisting of 2000 pulses. The waiting time between the trains of pulses varied between 1Hz and 10 Hz sessions to reach more matching stimulation time and to avoid potential overheating of the coil, as the DCC does not have a built-in cooling system. The participants were wearing earplugs during the rTMS session.” After reading this several times it is unclear to me how you stimulated. Did you stimulate with both 1Hz and 10Hz in the same session? If so how long did you wait between 1Hz and 10Hz? This is important information to consider carry over effects. This is clarified in your diagram, but may warrant clarification in the prose as well. When you say short trains what do you mean exactly? How long were the trains of 10Hz?, and were there trains of 1Hz, or was it delivered continuously? Did you alternate between trains of 1Hz and 10Hz? How did you choose the intertrain interval? Was it based on rMT? Or did you time match? I’m assuming there was no intertrain interval for the 1Hz stim, and this was just delivered continuously. Please consider clarifying. 

EEG analysis section: 
-Please consider clarifying this: “The electrodes were placed using the international 10-20 system with a reference electrode on the left mastoid.” When I read 10-20 system I assume you are hand measuring these placements. When I read high density EEG arrays I assume there is an EEG net that is started at specific points (eg. The nasion and inion), but largely conforms to the head without actually verifying the location of individual leads. 

Results/data section: 
-Please consider adding a brief section outlining the differences between your cannabis using and non-cannabis using participants. It appears there is a significant difference between groups in years of education, number of drinks/ and though non-significant, your non-using controls are older on average than your users (a larger sample, or a tighter distribution in your non-users would likely reveal a significant difference) 

Discussion: 
-You have a typo here “The aim of this study was to examine the role of the PCC and precuneus in processing external self-relevant stimuli in cannabis users and non-using controls by using rTMS to modulate activity in these region and assessing response to self-relevant non-drug and cannabis-related stimuli by measuring ERPs.” 

You may want to clarify whether this is normalized with HF or LF rTMS: “We found that cannabis users exhibited an increased response to self-relevant stimuli compared to controls during baseline that normalized after rTMS to the PCC and precuneus.” 


Is it possible that your findings are the result of group differences unrelated to cannabis use? Or that your findings are the result of subacute effect of cannabioniods (as the sobriety period is not that long, and verified by self-report only?). Obviously this cannot be controlled for retrospectively, but could be further discussed in your limitation section. 


Reviewer: 2 

Comments to the Author 
This is an interesting study on the influence of double-cone coil rTMS on brain structures involved in self-referential cognition, namely PCC and precuneus. Although the topic is highly relevant, the manuscript shows some major methodological flaws that need to be tackled before further consideration: 

1. You are mentioning that you used 1 Hz stimulation as a control condition for 10 Hz rTMS. To my knowledge, this is actually not a valid practice in state-of-the-art TMS application, since low- and high-frequency rTMS are considered to be both effectfull (Speer et al., 2014). Suggestion: since the authors have no valid control condition, they should only compare the pre- and post-stimulation sessions to each other and interpret the results separately based on current literature on high- and low-frequency rTMS efficacy. 

2. Based on your descriptions you also seem to have calculated ANOVAS separately for all the different task-conditions and electrodes and it is not clear what is meant when you say ‘baseline’, since there were actually two ‘baselines’ for each participant. It would be favorable if you would extend your description on data analysis. I would furthermore suggest a model like this: Factor 1 = group (user, non-user), Factor 2 = time-point (pre, post), Factor 3 = task-condition (oddball, self-relevant, cannabis-related). You should furthermore consider that the different ERPs at your electrodes of interest are not independent of each other and you should explain why you used exactly these electrodes for your analyses, since you had a cap with 64 electrodes. 

3. As you already mentioned yourselves, your sample size is to small to draw valid conclusions on cannabis users, especially since you have so many different conditions. We therefore suggest that you acquire another sample of 10 subjects per group in order to get appropriate results for the “EJN”. 


Speer, A.M., Wassermann, E.M., Benson, B.E., Herscovitch, P., Post, R.M., 2014. Antidepressant efficacy of high and low frequency rTMS at 110% of motor threshold versus sham stimulation over left prefrontal cortex. Brain Stimulation: Basic, Translational, and Clinical Research in Neuromodulation 7, 36-41. 

------------------------ 

____________________________________________________________________________________

Authors’ Response	02/08/2018

_____________________________________________________________________________________

We thank the expert reviewers for their constructive comments on this manuscript. The authors noted that the study was “highly relevant”, “well-designed and executed” and that the manuscript was “well-written”. The primary concerns involved the statistical approach, i.e., separate 2x3 ANOVA models for each stimulus, vs. an ANOVA model combining the stimuli types. In response, we re-analyzed our data using the suggested approach that led to overlapping findings with the original report. We have also clarified our methods and discussed the findings in context of the between group differences. We believe these modifications have improved the clarity of the manuscript, but we would be happy to make additional changes as necessary.

Please find our point-by-point response to the specific comments below. Modifications to the revised manuscript are underlined.

**Reviewer 1**

1. Please consider noting why you chose 4cm behind the motor strip. Is this a validated target? If not, how do you know you in fact hit precuneus/PCC?

RESPONSE: *This location (4 cm behind the motor strip) has been validated by Hayward et al., 2007 through rTMS and PET and also used by Vanneste, van der Loo, Plazier, & De Ridder, 2012. Both of these studies used the same double-cone coil used in the current study to target the PCC and found modulation of the PCC after rTMS. We have now added this information in the Methods Section.*

2. Please consider revising this section for clarity: *“Participants received repeated stimulation in random order at 1Hz and 10 Hz, each stimulation session consisting of 2000 pulses. The waiting time between the trains of pulses varied between 1Hz and 10 Hz sessions to reach more matching stimulation time and to avoid potential overheating of the coil, as the DCC does not have a built-in cooling system. The participants were wearing earplugs during the rTMS session.”* After reading this several times it is unclear to me how you stimulated. Did you stimulate with both 1Hz and 10Hz in the same session? If so how long did you wait between 1Hz and 10Hz? This is important information to consider carry over effects. This is clarified in your diagram, but may warrant clarification in the prose as well. When you say short trains what do you mean exactly? How long were the trains of 10Hz?, and were there trains of 1Hz, or was it delivered continuously? Did you alternate between trains of 1Hz and 10Hz? How did you choose the intertrain interval? Was it based on rMT? Or did you time match? I’m assuming there was no intertrain interval for the 1Hz stim, and this was just delivered continuously. Please consider clarifying.

RESPONSE: *We have added and adjusted the following information to the manuscript to clarify the rTMS administration:*

“The participants received repeated stimulation at 1Hz and 10 Hz in two randomized sessions with a one-week inter-session interval. The purpose of the one-week wash-out period was to avoid carry over effects (e.g., Schuwerk et al., 2014). Each rTMS session consisted of 2000 pulses. The 1Hz session consisted of 400 trains of 5 pulses in 5 seconds with an intertrain interval of 5 seconds, whereas the 10Hz session consisted of 40 trains of 50 pulses over 5 seconds with an intertrain interval of 50 seconds. The waiting time between the trains of pulses varied between the 1Hz and the 10 Hz session to reach more matching stimulation time and to avoid potential overheating of the coil, as the DCC does not have a built-in cooling system. The participants wore earplugs during the rTMS session.”

3. Please consider clarifying this: “The electrodes were placed using the international 10‐20 system with a reference electrode on the left mastoid.” When I read 10‐20 system I assume you are hand measuring these placements. When I read high density EEG arrays I assume there is an EEG net that is started at specific points (eg. The nasion and inion), but largely conforms to the head without actually verifying the location of individual leads.

RESPONSE: *We regret this confusion. We have clarified that we used an EEG net that had pre-configured electrodes according to the 10-20 international system and placed with reference to the nasion and inion:*

The electrodes were pre-configured in the EEG net according to the international 10-20 system and placed with reference to the nasion and inion.

4. Please consider adding a brief section outlining the differences between your cannabis using and non‐cannabis using participants. It appears there is a significant difference between groups in years of education, number of drinks/ and though non‐significant, your non‐using controls are older on average than your users (a larger sample, or a tighter distribution in your non‐users would likely reveal a significant difference)

RESPONSE: *Thank you for highlighting this important difference in our sample. We have outlined these differences in the results section:*

*“*There were no significant differences in age, gender, or ethnicity between the cannabis users and non-using controls; although, the controls as a group were older compared to the users. While there were no significant differences between number of alcohol drinking and smoking days in the preceding 90 days, on average the user group drank and smoked on more days (mean alcohol drinking days = 12.3 ± 12.8; mean smoking days = 17.9 ± 37.7) compared to the control group (mean alcohol drinking days = 5.7 ± 8.5; mean smoking days = 0.0 ± 0.0). The user group had significantly greater cannabis use compared to controls in the preceding 90 days (cannabis users mean = 76.7 ± 18.1 days; controls mean = 0.0 ± 0.0; *p* < 0.001). Two of the cannabis users met criteria for current cannabis abuse, five met criteria for lifetime cannabis abuse, one met criteria for current cannabis dependence, and three met criteria for lifetime cannabis dependence.”

*Additionally, we discussed the effect of these differences on our findings in the limitations section of the Discussion:*

“In addition, some results may reflect the differences between the groups in education, wider age distribution in the non-using control sample, and increased alcohol use in the cannabis-using group. A larger sample and tighter distribution may reveal additional differences in neural response.”

5. You have a typo here “The aim of this study was to examine the role of the PCC and precuneus in processing external self‐relevant stimuli in cannabis users and non‐using controls by using rTMS to modulate activity in these region and assessing response to self‐relevant non‐drug and cannabis‐related stimuli by measuring ERPs.”

RESPONSE: *Thank you for highlighting this typo. We have corrected the error as follows:*

“The aim of this study was to examine the role of the PCC and precuneus in processing external self-relevant stimuli in cannabis users and non-using controls. We used rTMS to modulate activity in these regions and assessed response to self-relevant and cannabis-related stimuli by recording ERPs.”

6. You may want to clarify whether this is normalized with HF or LF rTMS: “We found that cannabis users exhibited an increased response to self‐relevant stimuli compared to controls during baseline that normalized after rTMS to the PCC and precuneus.”

RESPONSE: *Thank you for highlighting this oversight. We have added that the response was normalized after HF rTMS:*

“We found that cannabis users exhibited an increased response to self-relevant stimuli compared to controls during baseline that normalized after HF rTMS to the PCC and precuneus.”

7. Is it possible that your findings are the result of group differences unrelated to cannabis use? Or that your findings are the result of subacute effect of cannabioniods (as the sobriety period is not that long, and verified by self‐report only?). Obviously this cannot be controlled for retrospectively, but could be further discussed in your limitation section.

RESPONSE: *Although we are limited in how much we can ascertain the underlying cause of these differences, we attempted to do so by correlating the amplitude and latency of the three components with (1) measures of cannabis use – SCID symptom count, MPS score, number of cannabis use days in the past 90 days, and (2) subacute effect of cannabinoids - hours since last use and THC metabolite levels. These correlations were not significant suggesting potential limitations of these measures in indicating subacute effects or that observed differences may be more nuanced. We have added this to the Discussion section:*

“There were no significant correlations between the amplitude and latency of the three components with measures of cannabis use, suggesting potential limitations of these measures in indicating subacute effects. Thus, it is unclear whether the results are a result of subacute effects of cannabinoids, the 24-hour abstinence period, or ongoing cannabis use and may be elucidated in future studies.”

**Reviewer 2**

1. You are mentioning that you used 1 Hz stimulation as a control condition for 10 Hz rTMS. To my knowledge, this is actually not a valid practice in state-of-the-art TMS application, since low- and high-frequency rTMS are considered to be both effectfull (Speer et al., 2014). Suggestion: since the authors have no valid control condition, they should only compare the pre- and post-stimulation sessions to each other and interpret the results separately based on current literature on high- and low-frequency rTMS efficacy.

RESPONSE: *This reviewer’s comment is well taken. In response, we have now conducted this analysis as suggested (described in response to the comment below). The overall results remained the same as the original report. The methods and results have been modified to reflect this change.*

2. Based on your descriptions you also seem to have calculated ANOVAS separately for all the different task conditions and electrodes and it is not clear what is meant when you say ‘baseline’, since there were actually two ‘baselines’ for each participant. It would be favorable if you would extend your description on data analysis. I would furthermore suggest a model like this: Factor 1 = group (user, non‐user), Factor 2 = time‐point (pre, post), Factor 3 = task‐condition (oddball, self‐relevant, cannabis‐related). You should furthermore consider that the different ERPs at your electrodes of interest are not independent of each other and you should explain why you used exactly these electrodes for your analyses, since you had a cap with 64 electrodes.

RESPONSE: *We appreciate this suggestion and have conducted the suggested ANOVA model instead comparing pre with post-LF rTMS and pre with post-HF rTMS separately. We additionally included region (frontal, central, parietal) and laterality (left, midline, right) as within subject factors to account for the dependence between the electrodes of interest. We have modified the description in the Methods section as follows:*

“A five-way mixed factorial (2x2x3x3x3) ANOVA with Group (cannabis user, non-using control) as the between subjects variable and rTMS Condition (baseline, LF rTMS), Stimulus (oddball, self-relevant, cannabis-related), Region (frontal, central, parietal), and Laterality (left, midline, right) as the within subject variables was conducted separately for the P3 component amplitude and latency. A separate five-way mixed factorial ANOVA was conducted to compare baseline and HF rTMS.”

*Unfortunately, we only have baseline data from the first session (before any rTMS administration). We have clarified this in the methods section and Figure 1:*

“In the first session, participants underwent EEG data collection during the task at a baseline session followed by rTMS administration in either the low frequency (LF; 1 Hz) or high frequency (HF; 10 Hz) condition. Participants repeated the EEG data collection during the modified oddball task after rTMS. In the second session, the participant underwent the LF or HF rTMS administration and the EEG data collection during the task. The LF and HF rTMS sessions were counter-balanced between the participants. This study design resulted in EEG data under three rTMS conditions: a baseline, after LF-rTMS, and after HF-rTMS.”

*We selected electrodes a priori based on the literature*. *The P3 component is largest in the centro-parietal electrodes (i.e., C3, Cz, C4, P3, Pz, and P4) (Johnson, Jr., 1993; Polich, 2007), the N2 component is greatest in the frontal electrodes (i.e., F3, Fz, and F4) (Anderer, Semlitsch, & Saletu, 1996; Novak, Ritter, & Vaughan, Jr., 1992), and the P2 component is greatest in the fronto-central electrodes (i.e., F3, Fz, F4, C3, Cz, and C4) (Anderer et al., 1996; Crowley & Colrain, 2004).* *We did not analyze all 64 electrodes as including sites where the ERP component is small results in additional noise (Luck, 2014). This a priori selection was consistent with our primary aim of examining changes in ERP response to stimuli before and after rTMS, rather than comparing differences in ERP distribution across the scalp. We focused on the conventional approach used to describe amplitude and latency to compare with previous studies. Further, to reduce Type I errors, we applied the very conservative Bonferroni correction for multiple comparisons, even for planned comparisons that were identified a priori.*

3. As you already mentioned yourselves, your sample size is to small to draw valid conclusions on cannabis users, especially since you have so many different conditions. We therefore suggest that you acquire another sample of 10 subjects per group in order to get appropriate results for the “EJN”.

RESPONSE: *To address the reviewer’s concern, we calculated the effect sizes and have now included them in the manuscript. The partial eta squared values ranged from 0.11 to 0.55 (mean = 0.24 ± 0.09), which are large effect sizes indicating that we had sufficient power to detect effects. In addition, we used a within-subjects design to reduce between-subject variability and implemented a randomized crossover study to counter-balance any order effects from the two rTMS conditions.*

*We also note that our sample size is appropriate for EJN (and other high impact journals) as indicated by recently published studies with similar approaches (within subjects design with both EEG and neuromodulation). As an example, we refer the reviewer to two recent papers in EJN: Bednar, Boland, & Lalor, 2017 (N=9) and de Taillez, Kollmeier, & Meyer, 2018 (N=10 per group). Others include Brain Stimulation (Angius et al., 2018; N=12), Journal of Neuroscience (Keller et al., 2018; N=8 per group), NeuroImage (Pisoni, Romero Lauro, Vergallito, Maddaluno, & Bolognini, 2018; N=11 per group).*

*Regardless, we emphasize the need to replicate and extend these findings in the limitations section.*

References

Anderer, P., Semlitsch, H. V., & Saletu, B. (1996). Multichannel auditory event-related brain potentials: effects of normal aging on the scalp distribution of N1, P2, N2 and P300 latencies and amplitudes. *Electroencephalography and Clinical Neurophysiology*, *99*, 458–472.

Angius, L., Mauger, A. R., Hopker, J., Pascual-Leone, A., Santarnecchi, E., & Marcora, S. M. (2018). Bilateral extracephalic transcranial direct current stimulation improves endurance performance in healthy individuals. *Brain Stimulation*, *11*, 108–117.

Bednar, A., Boland, F. M., & Lalor, E. C. (2017). Different spatio-temporal electroencephalography features drive the successful decoding of binaural and monaural cues for sound localization. *European Journal of Neuroscience*, *45*, 679–689.

Crowley, K. E., & Colrain, I. M. (2004). A review of the evidence for P2 being an independent component process: age, sleep and modality. *Clinical Neurophysiology*, *115*, 732–744.

de Taillez, T., Kollmeier, B., & Meyer, B. T. (2018). Machine learning for decoding listeners’ attention from electroencephalography evoked by continuous speech. *European Journal of Neuroscience*. doi:10.1111/ejn.13790

Hayward, G., Mehta, M. A., Harmer, C., Spinks, T. J., Grasby, P. M., & Goodwin, G. M. (2007). Exploring the physiological effects of double-cone coil TMS over the medial frontal cortex on the anterior cingulate cortex: an H 2 15 O PET study. *European Journal of Neuroscience*, *25*, 2224–2233.

Johnson, Jr., R. (1993). On the neural generators of the P300 component of the event-related potential. *Psychophysiology*, *30*, 90–97.

Keller, C. J., Huang, Y., Herrero, J. L., Fini, M., Du, V., Lado, F. A., … Mehta, A. D. (2018). Induction and quantification of excitability changes in human cortical networks. *The Journal of Neuroscience*, 1088–17.

Luck, S. J. (2014). *An Introduction to the Event-Related Potential Technique* (2nd ed.). Cambridge, MA: MIT Press.

Novak, G., Ritter, W., & Vaughan, Jr., H. G. (1992). Mismatch detection and the latency of temporal judgments. *Psychophysiology*, *29*, 398–411.

Pisoni, A., Romero Lauro, L. J., Vergallito, A., Maddaluno, O., & Bolognini, N. (2018). Cortical dynamics underpinning the self-other distinction of touch: A TMS-EEG study. *NeuroImage*, *178*, 475–484.

Polich, J. (2007). Updating P300: An integrative theory of P3a and P3b. *Clinical Neurophysiology*, *118*, 2128–2148.

Vanneste, S., van der Loo, E., Plazier, M., & De Ridder, D. (2012). Parietal double-cone coil stimulation in tinnitus. *Experimental Brain Research*, *221*, 337–343.

2^nd^ Editorial Decision	24/08/2018

_____________________________________________________________________________________

Testing the role of the posterior cingulate cortex in processing salient stimuli in cannabis users: an rTMS study 

Dear Dr. Filbey, 

Your revised manuscript was re-evaluated by external reviewers as well as by the Section Editor, Dr. Rita Goldstein and ourselves.  We are pleased to inform you that it will be accepted for publication in EJN pending a few mior revisions (see below).  Once done, we will send it off to production.   

- Please indicate ethical approval from institutional body. 
- Please indicate in the data statement to whom should requests be made. 
- The small figures (2-5) will need to be enlarged for publication. 
- '..controls mean = 0.0 ± 0.0...'.  This occurs in several places and is not 'scientific'. 
- Please include precise P values in the legends. 

When revising the manuscript, please embolden or underline major changes to the text so they are easily identifiable and please don't leave 'track change' formatting marks in your paper.  At this stage, please provide text and a figure file for the Graphical Abstract.  When carrying out your revisions please refer to the checklist below and visit the EJN author guidelines at www.ejneuroscience.org 

When finalised, please upload your complete revised manuscript onto the website as a Word (.doc or .docx), or .rtf file.  Please also ensure that a complete set of tables and figures is included as separate files, even if these have not changed from the originals.  At this stage it is necessary to provide high resolution figures.  Please see important instructions below. 

Please go into https://mc.manuscriptcentral.com/ejn - Author Centre - manuscripts with decisions where you will find a 'create a revision' link under 'actions'. We ask that you please indicate the way in which you have responded to the points raised by the Editors and Reviewers in a letter. Please upload this response letter as a separate Word (.doc) file using the file designation "Authors' Response to Reviewers" when uploading your manuscript files. Please DO NOT submit your revised manuscript as a new one.  Also, please note that only the  Author who submitted the original version of the manuscript should submit a revised version. 

If you are able to respond fully to the points raised, we shall be pleased to receive a revision of your paper within 30 days. 

Thank you for your support of this Special Issue of EJN. 

Best wishes, 

Paul Bolam & John Foxe 
co-Editors in Chief, EJN 

Reviews: 

Reviewer: 2 

Comments to the Author 
The authors sufficiently addressed most of my comments. I appreciate the efforts undertaken and think reporting the findings obtained and the limitations due to limited sample size in the proposed study beiing valuable. Therefore I endorse the publication of this manuscript. 

I want to take the opportunity to bring an article to the author's awareness, that we published in Neuroimage, http://dx.doi.org/10.1016/j.neuroimage.2017.09.022 . There we highlight a resting-state network (RSN#17 from 1000 Functional Connectomes) with specific modulations after rTMS over one of its encompassing regions (DLPFC). We have recently acquired preliminary data demonstrating that stimulating another region of the same network (TPJ) again modulates the same network. Since your stimulation target is also part of this meso-cortico-limbic network, the effects you observe could be explained due to network modulations within it. 

Best, Martin. 

Reviewer: 1 

Comments to the Author 
My concerns have been addressed. 

------------------------ 

_____________________________________________________________________________________

Authors’ Response	11/09/2018

_____________________________________________________________________________________

Thank you for accepting this manuscript for publication. We have addressed the minor revisions below. Modifications to the revised manuscript are underlined.

1. Please indicate ethical approval from institutional body.

RESPONSE: We have updated the methods section to include this statement: “All participants provided written informed in accordance with World Medical Association Declaration of Helsinki and the research protocol was approved by the Institutional Review Board (IRB) of the University of Texas at Dallas.”

2. Please indicate in the data statement to whom should requests be made.

REPONSE: We have updated the data statement: “The data are available upon request to Francesca Filbey.”

3. The small figures (2-5) will need to be enlarged for publication.

RESPONSE: We have enlarged Figures 2-5.

4. '..controls mean = 0.0 ± 0.0...'. This occurs in several places and is not 'scientific'.

RESPONSE: We have changed this to “controls mean = 0”. For these variables, all individuals in the group had a value of 0.

5. Please include precise P values in the legends.

RESPONSE: We have added the *p*-values to the legends.
